# Quality of life among earthquake survivors living in prefabricated houses in Adıyaman: a cross-sectional study after the February 2023 earthquakes in Türkiye

**DOI:** 10.3389/fpubh.2025.1567881

**Published:** 2025-04-10

**Authors:** Yusuf Emre Bostan, Yusuf Demirtaş

**Affiliations:** ^1^Adıyaman Provincial Health Directorate, Adıyaman, Türkiye; ^2^Department of Public Health, Faculty of Medicine, Ordu University, Ordu, Türkiye

**Keywords:** quality of life, earthquake, disaster, Turkey, survivors, temporary housing, SF-36

## Abstract

**Introduction:**

On February 6, 2023, two devastating earthquakes in Türkiye caused significant loss of life and widespread destruction, forcing many survivors into temporary housing. The earthquakes have the potential to significantly impact the quality of life of survivors, exacerbating various dimensions of their physical, emotional, and social well-being. This study aims to assess quality of life among earthquake survivors residing in prefabricated housing in Adıyaman, one of the most severely affected provinces, and to identify associated factors.

**Methods:**

This cross-sectional study was conducted with 334 adult earthquake survivors residing in prefabricated housing in Adıyaman. Socio-demographic earthquake-related characteristics were recorded, and quality of life was assessed using the Short Form-36 (SF-36) through face-to-face interviews. The relationship between quality of life and independent variables was analyzed using the Mann–Whitney U test and multivariate logistic regression analysis.

**Results:**

All eight domains of the SF-36 showed a decline in comparison to general population norms. The smallest decrease was observed in the ‘physical functioning’ domain (7.6% in men and 15.4% in women), while the largest decline occurred in the ‘role limitations due to emotional problems’ domain (32.1% in men and 45.6% in women). Female gender, loss of a relative, hospitalization due to the earthquake, being married, being over 35 years old and having an education level below high school were identified as risk factors for scoring below the general population norms in at least one domain of the SF-36.

**Conclusion:**

Sixteen months after the earthquake, the quality of life among survivors remains significantly low, highlighting the critical need for the rapid implementation of targeted interventions, prioritizing high-risk groups.

## 1 Introduction

Earthquakes are widespread natural disasters that occur globally. However, large-scale earthquakes can pose severe threats, resulting in significant trauma and life-threatening consequences for individuals and communities (1). Their unpredictability and destructive impacts can lead to serious adverse effects on both physical and mental health (2).

On February 6, 2023, two major earthquakes struck the Kahramanmaraş province in Türkiye. The first earthquake, with a magnitude of 7.7 on the Richter scale, occurred in the Pazarcık district at 04:17 a.m., followed by a second earthquake of 7.6 magnitude in the Elbistan district at 1:24 p.m. the same day. These earthquakes, felt across 11 cities, caused catastrophic loss of life and extensive structural damage. Official reports indicate over 50,000 deaths and more than 115,000 injuries, making this disaster one of the most severe in Türkiye’s history. Due to their massive scale and impact, these events have been referred to as the “disaster of the century.” The occurrence of two powerful earthquakes within a nine-hour interval intensified the destruction, leading to substantial negative impacts on the survivors (3, 4).

Adıyaman province, which borders Kahramanmaraş to the east, was one of the most severely affected regions. It was reported that 8,327 people died in Adıyaman due to the earthquake (5). Thousands of buildings in the city were either destroyed or severely damaged, creating a significant housing crisis for the survivors. As a result, many individuals had to live in prefabricated houses within temporary shelter areas (6).

Many studies have primarily focused on the mental health impacts of earthquakes on survivors. However, the effects of earthquakes on health are not limited to mental health. Research has shown that earthquakes can significantly affect the quality of life (QoL) of survivors (1, 7). The World Health Organization defines QoL as “individuals’ perception of their position in life in the context of the culture and value systems in which they live, and in relation to their goals, expectations, standards, and concerns” (8). Assessing QoL involves examining the physical, cognitive, emotional, and sociological dimensions of life, enabling a thorough and multidimensional analysis of risk factors linked to the impacts of earthquakes across these critical domains (1). In addition to the direct effects of the earthquake, post-earthquake extraordinary housing conditions may contribute to notable declines in various dimensions of individuals’ well-being (7). Given this broad scope, evaluating QoL offers a critical lens to comprehensively understand the multidimensional consequences of disasters on affected populations. QoL evaluations following a disaster are also valuable in providing evidence for monitoring and assessing interventions related to societal recovery efforts (9).

Studies have identified several factors associated with poor QoL among earthquake survivors. Variables such as age, gender, education level, economic challenges, physical illnesses, and mental health conditions have been found to influence QoL. Moreover, these associations may vary across affected populations (10–12). Identifying these factors among survivors is crucial for determining risk groups and guiding targeted interventions.

The objective of this study was to assess the QoL among earthquake survivors residing in prefabricated housing in Adıyaman following the February 2023 earthquakes and to identify the factors that are associated with QoL.

## 2 Materials and methods

### 2.1 Study design and participants

This cross-sectional study was conducted between July 1, 2024, and August 10, 2024, among adult earthquake survivors residing in prefabricated housing in Adıyaman. A prefabricated housing area, selected from among those located in different parts of the city center, was designated as the study site. The selected area accommodates a total of 600 earthquake survivors. Although the exact size of the adult population in the area is unknown, it is estimated that the target population comprises approximately 403 individuals, based on the percentage of adults in the overall population of Adıyaman. Individuals aged 18 years or older, who had experienced the earthquake, were residing in prefabricated housing, and were able to communicate, were invited to participate in the study. A total of 334 participants who completed the survey were included in the study (82.9% of the estimated target population).

### 2.2 Data collection and measurement tools

The research data were collected through a survey administered via face-to-face interviews. The survey was conducted by trained healthcare professionals, in accordance with the study protocol. The survey consisted of two sections. The first section included questions related to participants’ socio-demographic characteristics and earthquake-related attributes. Information including age, gender, marital status, education level, monthly household income, hospitalization due to the earthquake, and loss of relatives as a result of the earthquake was recorded. The second section comprised the Short Form-36 (SF-36), a tool designed to evaluate QoL. The SF-36 consists of 36 items organized into eight domains: physical functioning (PF), social functioning (SF), role limitations due to physical health problems (RP), role limitations due to emotional problems (RE), mental health (MH), vitality (VT), bodily pain (BP), and general health perceptions (GH). Each domain is scored on a scale of 0 to 100, with higher scores indicating better QoL (13). The Turkish version of the SF-36 has been demonstrated to have strong reliability and validity and has been widely used in numerous studies (14). Fifty-three participants did not provide data on monthly household income, and thus, the analysis related to income was conducted using data from 281 participants. Data for all other variables were available for all 334 participants.

### 2.3 Statistical analysis

The quantitative data were presented as median and interquartile range (IQR), while categorical data were expressed as frequency and percentage. The SF-36 domain scores of participants were presented separately for women and men, expressed as both mean (SD) and median (IQR), and for each domain, the percentage change from the general population norm was calculated using the following formula: ((B/A – 1) × 100), where A represents the mean domain score of the participants, and B represents the mean domain score of the general population norm. The normality of the quantitative data distribution was assessed using the Shapiro–Wilk test. Since none of the eight domain scores followed a normal distribution, the relationship between participants’ SF-36 domain scores and age group, gender, marital status, education level, monthly household income group, loss of relatives, and hospitalization due to the earthquake was evaluated using the Mann–Whitney U test. A further analysis was conducted by dichotomizing participants based on whether their domain scores were below the general population norm or equal to/above it, stratified by gender. To account for potential confounding effects and evaluate the independent associations of multiple variables with QoL outcomes, backward multivariate logistic regression analysis was performed to systematically refine the model and identify the most relevant factors associated with each SF-36 domain score falling below the general population norm. Initially, all six candidate independent variables—age group, gender, marital status, education level, loss of relatives, and hospitalization due to the earthquake—were entered into the model for each domain of the SF-36. Due to a significant association between education level and monthly household income, monthly household income was excluded from the analysis at the outset to prevent multicollinearity. The association between the outcome and the final set of variables was assessed by calculating odds ratios (OR) with 95% confidence intervals (CI). Statistical analyses were performed using JASP (version 0.18.3, University of Amsterdam, Netherlands). Specifically, the Shapiro–Wilk test for normality was applied to each of the eight SF-36 domains using the “Descriptives” module to assess the distribution of the data. The Mann–Whitney U test was performed using the “T-Tests” module, selecting the Mann–Whitney option to evaluate the univariate associations between each SF-36 domain score and the dichotomized independent variables. To assess the independent effects of multiple variables on QoL outcomes, eight separate multivariate logistic regression analyses were conducted—one for each SF-36 domain. These analyses were performed using the “Regression” module, selecting the “Logistic Regression” option with the backward stepwise method enabled to systematically refine each model. A *p*-value of less than 0.05 was considered statistically significant.

## 3 Results

The study was conducted with a total of 334 participants. The mean age of the participants was 36.2 ± 11.3 years, and 50.9% were female. The majority of the participants were married, and most had an education level lower than high school. A total of 28.4% had lost at least one relative due to the earthquake, and 7.5% had been hospitalized as a result of the earthquake. The general characteristics of the study population are presented in Table 1.

**Table 1:** General characteristics of the study population (*n* = 334).

Characteristics	*n*	%
Age (mean ± SD)	36.2 ± 11.3	
Gender		
Male	164	49.1
Female	170	50.9
Marital status		
Married	223	66.8
Single	96	28.7
Divorced/Widowed	15	4.5
Education level		
No degree	22	6.6
Below high school	157	47.0
High school	140	41.9
Above high school	15	4.5
Monthly household income (TRY, mean ± SD)	35719.8 ± 27073.4	
Loss of relatives		
Yes	95	28.4
No	239	71.6
Hospitalization due to earthquake		
Yes	25	7.5
No	309	92.5

SD, Standard Deviation; TRY, Turkish Lira.

Among earthquake survivors of both genders, the mean scores for all eight domains of the SF-36 were lower than the general population norms. The smallest decline was observed in the physical functioning (PF) domain among men (7.59%), while the largest decline was in the role limitations due to emotional problems (RE) domain among women (45.58%). The SF-36 scores of the respondents and the general population norms are presented in Table 2.

**Table 2:** SF-36 scores of the respondents and general population norms.

SF-36 Domains	Men				Women			
	Respondents (*n* = 164)		Norms of general population (*n* = 609)*	Decrease in mean	Respondents (*n* = 170)		Norms of general population (*n* = 670)*	Decrease in mean
	Median (IQR)	Mean ± SD	Mean ± SD	%	Median (IQR)	Mean ± SD	Mean ± SD	%
PF	90.0 (65.0–100.0)	80.58 ± 22.56	87.2 ± 17.1	7.59	70.0 (50.0–90.0)	68.21 ± 25.63	80.6 ± 21.7	15.37
RP	75.0 (25.0–100.0)	67.07 ± 38.55	89.8 ± 19.3	25.31	50.0 (25.0–100.0)	53.09 ± 37.01	82.9 ± 28.6	35.96
GH	50.0 (40.0–65.0)	52.26 ± 20.53	73.6 ± 14.9	28.99	45.0 (35.0–55.0)	44.74 ± 16.69	69.1 ± 16.9	35.25
BP	70.0 (54.4–90.0)	69.35 ± 22.81	85.1 ± 16.4	18.51	57.5 (45.0–77.5)	57.91 ± 24.36	81.0 ± 20.2	28.51
VT	50.0 (35.0–60.0)	47.29 ± 19.47	65.7 ± 11.9	28.02	45.0 (25.0–50.0)	40.32 ± 19.39	63.4 ± 13.7	36.40
SF	62.5 (50.0–75.0)	64.18 ± 22.18	91.7 ± 12.8	30.01	50.0 (50.0–75.0)	57.79 ± 24.09	90.1 ± 12.9	35.86
RE	66.7 (33.3–100.0)	63.01 ± 40.95	92.8 ± 15.1	32.10	33.3 (0.0–100.0)	48.43 ± 41.51	89.0 ± 22.5	45.58
MH	52.0 (40.0–60.0)	50.97 ± 17.17	71.0 ± 10.6	28.21	52.0 (36.0–60.0)	48.40 ± 17.56	70.1 ± 11.4	30.96

* Norms for the general population in Türkiye by gender were obtained from the study conducted by Demiral et al. (16).

PF, Physical Functioning; RP, Role Limitations Due to Physical Health Problems; GH, General Health Perceptions; BP, Bodily Pain; VT, Vitality; SF, Social Functioning; RE, Role Limitations Due to Emotional Problems; MH, Mental Health; SD, Standard Deviation; IQR, Interquartile Range.

The SF-36 scores of respondents based on sociodemographic and earthquake-related factors are presented in Table 3. Participants who were hospitalized due to the earthquake had significantly lower scores across all SF-36 domains compared to those who were not hospitalized. Being female was associated with lower scores in all domains except for MH. The loss of relatives was linked to lower scores in the PF, GH, BP, SF, and RE domains. Similarly, lower monthly household income was associated with lower scores in the PF, RP, GH, BP, and RE domains. Additionally, participants aged over 35, those who were married, and those with an education level below high school had lower scores in multiple SF-36 domains.

**Table 3:** SF-36 scores of the respondents by sociodemographic and earthquake-related factors, median (interquartile range).

	SF-36 Domains							
Independent variables	PF	RP	GH	BP	VT	SF	RE	MH
Age								
≤35	85.0 (63.8–100.0)	75.0 (25.0–100.0)	50.0 (40.0–65.0)	67.5 (45.0–80.0)	50.0 (35.0–55.0)	62.5 (50.0–75.0)	66.7 (25.0–100.0)	52.0 (39.0–60.0)
>35	75.0 (50.0–93.8)	62.5 (25.0–100.0)	45.0 (35.0–55.0)	57.5 (45.0–77.5)	45.0 (25.0–50.0)	62.5 (50.0–75.0)	66.7 (0.0–100.0)	52.0 (37.0–59.0)
*p*	*<0.001*	*NS*	*<0.001*	*NS*	*0.003*	*NS*	*NS*	*NS*
Gender								
Male	90.0 (65.0–100.0)	75.0 (25.0–100.0)	50.0 (40.0–65.0)	70.0 (54.4–90.0)	50.0 (35.0–60.0)	62.5 (50.0–75.0)	66.7 (33.3–100.0)	52.0 (40.0–60.0)
Female	70.0 (50.0–90.0)	50.0 (25.0–100.0)	45.0 (35.0–55.0)	57.5 (45.0–77.5)	45.0 (25.0–50.0)	50.0 (50.0–75.0)	33.3 (0.0–100.0)	52.0 (36.0–60.0)
*p*	*<0.001*	*<0.001*	*<0.001*	*<0.001*	*0.002*	*0.009*	*0.002*	*NS*
Marital status								
Married	75.0 (55.0–95.0)	50.0 (25.0–100.0)	45.0 (35.0–55.0)	57.5 (45.0–77.5)	45.0 (30.0–50.0)	62.5 (50.0–75.0)	66.7 (0.0–100.0)	52.0 (40.0–56.0)
Single/Divorced/Widowed	90.0 (60.0–100.0)	75.0 (25.0–100.0)	50.0 (40.0–65.0)	67.5 (50.0–83.8)	50.0 (35.0–55.0)	62.5 (50.0–87.5)	66.7 (33.3–100.0)	52.0 (36.0–64.0)
*p*	*<0.001*	*NS*	*0.010*	*0.026*	*0.038*	*NS*	*NS*	*NS*
Education level								
Below High School	65.0 (50.0–90.0)	50.0 (25.0–100.0)	45.0 (35.0–55.0)	57.5 (45.0–77.5)	50.0 (30.0–52.5)	62.5 (50.0–75.0)	66.7 (0.0–100.0)	52.0 (44.0–60.0)
High School and Above	90.0 (70.0–100.0)	75.0 (25.0–100.0)	50.0 (40.0–65.0)	67.5 (45.0–80.0)	45.0 (30.0–52.5)	62.5 (50.0–75.0)	66.7 (33.3–100.0)	52.0 (36.0–60.0)
*p*	*<0.001*	*NS*	*0.003*	*0.033*	*NS*	*NS*	*NS*	*NS*
Monthly Household Income								
<30,000 TRY	70.0 (50.0–90.0)	50.0 (25.0–100.0)	45.0 (35.0–56.3)	57.5 (45.0–77.5)	45.0 (30.0–50.0)	62.5 (50.0–75.0)	66.7 (0.0–100.0)	52.0 (40.0–56.0)
≥30,000 TRY	90.0 (65.0–100.0)	75.0 (25.0–100.0)	50.0 (40.0–65.0)	67.5 (45.0–80.0)	45.0 (30.0–55.0)	62.5 (50.0–75.0)	66.7 (33.3–100.0)	52.0 (36.0–60.0)
*p*	*<0.001*	*0.036*	*0.035*	*0.027*	*NS*	*NS*	*0.026*	*NS*
Loss of relatives								
Yes	65.0 (50.0–85.0)	50.0 (25.0–100.0)	45.0 (35.0–52.5)	55.0 (45.0–67.5)	45.0 (25.0–55.0)	50.0 (50.0–75.0)	33.3 (0.0–100.0)	48.0 (36.0–56.0)
No	85.0 (60.0–100.0)	75.0 (25.0–100.0)	50.0 (40.0–60.0)	67.5 (45.0–80.0)	50.0 (30.0–50.0)	62.5 (50.0–75.0)	66.7 (33.3–100.0)	52.0 (40.0–60.0)
*p*	*<0.001*	*NS*	*0.005*	*<0.001*	*NS*	*0.002*	*0.003*	*NS*
Hospitalization due to earthquake								
Yes	55.0 (45.0–80.0)	0.0 (0.0–75.0)	40.0 (30.0–50.0)	45.0 (45.0–57.5)	30.0 (20.0–50.0)	50.0 (12.5–62.5)	0.0 (0.0–33.3)	44.0 (36.0–56.0)
No	85.0 (60.0–100.0)	75.0 (25.0–100.0)	50.0 (40.0–60.0)	67.5 (45.0–80.0)	50.0 (30.0–55.0)	62.5 (50.0–75.0)	66.7 (33.3–100.0)	52.0 (40.0–60.0)
*p*	*0.003*	*<0.001*	*0.006*	*0.003*	*0.003*	*<0.001*	*<0.001*	*0.048*

PF, Physical Functioning; RP, Role Limitations Due to Physical Health Problems; GH, General Health Perceptions; BP, Bodily Pain; VT, Vitality; SF, Social Functioning; RE, Role Limitations Due to Emotional Problems; MH, Mental Health; TRY, Turkish Lira; NS, Not significant; (*p* > 0.05).

Multivariate logistic regression analysis identified several sociodemographic and earthquake-related factors associated with scoring below the general population norms in SF-36 domains (Table 4). The risk of scoring below the general population norm in PF was higher among respondents over 35 years old, female participants, those who lost a relative in the earthquake, and those who were hospitalized, while it was lower among individuals with at least a high school education. In RP, this risk was higher among those of female gender and those who were hospitalized. In VT, female gender increased the risk, while in SF, losing a relative was a risk factor. In BP, both female gender and losing a relative were associated with a higher risk. In GH, the risk was lower among those who were not married and those with at least a high school education, while in MH, it was lower among those who were not married. The risk of scoring below the general population norm in RE was higher among female participants, those who lost a relative, and those who were hospitalized.

**Table 4:** Multivariate logistic regression analysis of factors associated with scoring below general population norms on SF-36 domains by gender, odds ratio (95% confidence interval).

	SF-36 Domains							
Independent variables	PF	RP	GH	BP	VT	SF	RE	MH
Age (Ref: ≤35)								
>35	1.70* (1.02–2.86)	*Exc.*	*Exc.*	*Exc.*	*NS*	*Exc.*	*Exc.*	*Exc.*
Gender (Ref: Male)								
Female	1.65* (1.02–2.68)	2.68** (1.69–4.25)	*Exc.*	2.28** (1.30–3.99)	2.52** (1.28–4.98)	*Exc.*	1.89** (1.20–2.97)	*NS*
Marital status (Ref: Married)								
Single/Divorced/Widowed	*NS*	*Exc.*	0.36** (0.18–0.70)	*Exc.*	*Exc.*	*Exc.*	*Exc.*	0.47* (0.22–0.98)
Education level (Ref: Below High School)								
High School and Above	0.38** (0.23–0.61)	*Exc.*	0.38** (0.19–0.76)	*Exc.*	*Exc.*	*Exc.*	*Exc.*	*Exc.*
Loss of relatives (Ref: No)								
Yes	2.72** (1.56–4.74)	*Exc.*	*Exc.*	2.59* (1.25–5.37)	*Exc.*	4.91* (1.47–16.43)	1.84* (1.08–3.11)	*NS*
Hospitalization due to earthquake (Ref: No)								
Yes	2.96* (1.01–8.66)	3.53* (1.16–10.73)	*Exc.*	*NS*	*NS*	*Exc.*	3.40* (1.12–10.37)	*Exc.*

*p < 0.05; **p < 0.01.

PF, Physical Functioning; RP, Role Limitations Due to Physical Health Problems; GH, General Health Perceptions; BP, Bodily Pain; VT, Vitality; SF, Social Functioning; RE, Role Limitations Due to Emotional Problems; MH, Mental Health; Exc., Excluded during backward elimination; NS, Not significant; (*p* > 0.05).

## 4 Discussion

Earthquakes can adversely affect the health and well-being of survivors, leading to physical and psychological challenges that diminish their QoL. Our study found that earthquake survivors residing in prefabricated housing in Adıyaman experienced a decline in all SF-36 domains compared to general population norms. Additionally, each of the examined variables—age, gender, marital status, education level, monthly household income, loss of relatives, and hospitalization due to the earthquake—was associated with an increased risk of scoring below the population norm in at least one SF-36 domain.

The worsening of QoL among earthquake survivors has been reported in various populations across several studies. Following the 8.0 magnitude earthquake in Wenchuan, China, in 2008, Ke et al. used the same instrument as our study to assess the QoL of participants. They found that participants had lower scores than the general population norms across all domains, 8 months after the earthquake (7). Similarly, in a study conducted by Yabuki et al., older adult survivors residing in temporary housing after the 2011 Great East Japan Earthquake were assessed using the SF-36. They reported that six out of the eight domains had significantly lower scores when compared to national standards, with the exceptions of bodily pain and vitality (15). Our study population closely mirrors national statistics in terms of age and gender, with the median age of Türkiye’s population being 34 years and the proportion of females at 49.9%. According to the SF-36 norms established by Demiral et al. for the general population, a decline in scores was observed across all domains for both male and female participants (16–18). Notably, the reductions in domain scores observed in our study are more pronounced compared to those reported by Ke et al., with the reductions ranging from 4.5 to 19.6% in their study, representing the lowest and highest declines across different domains (7). Given that our respondents were earthquake survivors residing in prefabricated housing, coupled with the physical, mental, and social challenges associated with prolonged displacement, it is likely that these factors contributed to a more pronounced decline in their QoL. Moreover, variations in post-earthquake recovery strategies, regional resilience levels, and cultural factors may have significantly shaped the differences observed in QoL outcomes.

The Physical Functioning (PF) domain demonstrated the least decline in comparison to population norms for both males and females. This could primarily be attributed to the fact that the average age of our study population falls within the prime working years, a period typically associated with greater physical resilience. The most significant decline in both genders, however, was observed in the Role Limitations Due to Emotional Problems (RE) domain. This demonstrates the significant impact of earthquake-related psychological trauma, which appears to impair individuals’ ability to fulfill personal, professional, and social roles, thereby emphasizing the intricate relationship between emotional well-being and daily functioning in the aftermath of a disaster. Such disruptions highlight the need for comprehensive support strategies to foster emotional recovery and restore functional well-being among survivors. Interestingly, the decrease in the Mental Health (MH) domain was less pronounced compared to the Role Limitations Due to Emotional Problems (RE) domain. This discrepancy may be attributed to cultural and social resilience factors, as mental health issues are often more internal and may be better managed through these mechanisms. In contrast, Role Limitations Due to Emotional Problems (RE), which directly impacts daily role performance, requires more external expression, and the post-earthquake stress and trauma may have had a more significant effect on this domain. Given the living conditions of our study population, it is plausible that environmental factors have exacerbated role limitations. Therefore, this discrepancy warrants further investigation.

Numerous studies have reported that post-earthquake QoL tends to be lower among women compared to men. Following the 7.3-magnitude Chi-Chi earthquake in Taiwan, Tsai et al. found that scores in the General Health (GH), Vitality (VT), and Mental Health (MH) domains of the SF-36 were negatively correlated with female gender even 3 years after the disaster (19). Similarly, a study conducted 2 years after the 7.0-magnitude Jiuzhaigou earthquake in China identified female gender as a risk factor for the Physical Component Summary (PCS) of the SF-12, though no significant association was found with the Mental Component Summary (MCS) (20). In our study, women had significantly lower scores across all SF-36 domains compared to men, except for the MH domain. However, some studies have reported no significant association between post-earthquake QoL and gender (11, 12). Various factors, including the severity of the earthquake, characteristics of the affected population, the time elapsed since the disaster, and living conditions, may influence these discrepancies. Nevertheless, our findings suggest that women represent a key risk group for post-earthquake deterioration in QoL.

In our study, respondents with a history of hospitalization due to the earthquake had lower QoL scores, as expected. Similar associations have been reported in various QoL studies involving earthquake survivors who were hospitalized or injured (12, 20). However, the fact that significant declines were observed across all SF-36 domains in our study is particularly noteworthy. Given that our study was conducted 16 months after the earthquake, the persistence of these impairments highlights the long-term impact of earthquake-related injuries and underscores the critical need for sustained medical and psychosocial support for this vulnerable group.

In addition to gender and earthquake-related hospitalization, various sociodemographic and disaster-related factors were associated with declines in certain QoL domains. The most prominent among these were the loss of relatives and low monthly household income, both linked to lower scores in five different domains, reflecting the burden of bereavement and financial hardship. Additionally, older age, being married, and lower educational attainment were also associated with lower scores across multiple domains.

Although the method of assessing the relationship between independent variables and the QoL scores of earthquake victims, as used in most studies, is an important indicator, it is an approach that ignores community norms. Therefore, we conducted a multivariable logistic regression analysis to assess the risk of individuals falling below the general population norms. This approach allowed us to comprehensively examine the determinants of deteriorating QoL, not only within the participants’ own group but also in relation to national standards. The logistic regression analysis revealed that the risk of falling below the general population norms remained statistically significant for at least one domain across all variables included in the model, highlighting their independent contributions to post-earthquake QoL. Being female was the variable most frequently associated with scores below the general population norms across domains. Similarly, loss of relatives was found to be associated with scores below the standards in half of the domains, underscoring the need for targeted interventions focusing on grief counseling.

There are studies reporting a negative relationship between factors such as older age, earthquake-related injury, low household income, and loss or injury of relatives with the Physical Functioning (PF) domain scores in multivariable analyses (10, 12). In our study, older age, female gender, lower educational level, loss of relatives, and hospitalization due to the earthquake were found to be associated with a higher risk of having PF scores below the general population norms. As for Role Limitations Due to Physical Health Problems (RP), the risk was found to be higher among women and those who were hospitalized, while for Role Limitations Due to Emotional Problems (RE), a similar pattern emerged, with loss of relatives also being identified as a significant factor, as might be anticipated. However, it was surprising that only being married was found to be associated with scores below the general population norms in the Mental Health (MH) domain. Wu et al. reported that, 3 years after the earthquake in Taiwan, being married was associated with higher MH scores among earthquake survivors. Similarly, male gender and better financial status were also found to be associated with higher scores (10). Considering that being married is generally associated with positive effects such as providing social support and aiding in coping with stress, this finding is unexpected. A similar finding to our study regarding marital status was reported in the study by Chen et al., which examined QoL among disaster relief volunteers and highlighted the burdens associated with marital responsibilities (21). Disasters may lead to changes in family dynamics and structure. The traditionally protective role of marriage may be altered in disaster-affected communities due to the increased stress and responsibilities within family life (22). Furthermore, a large-scale study assessing the relationship between marital status and QoL using EQ-VAS in a multilevel analysis found that being single was associated with higher QoL, particularly among younger adults (23). The relatively young age of our study population may have influenced our findings. The interplay of factors such as the characteristics of the study population, cultural context, and the increased pressures arising from shifts in family roles and responsibilities due to the earthquake likely played a role in shaping this outcome. Regarding the Social Functioning (SF) domain, the risk was elevated only among those who had lost relatives, whereas for the Vitality (VT) domain, it was higher solely among women. In the case of the Bodily Pain (BP) domain, both factors were associated with an increased risk. The varying degrees of impact across different domains underscore the complex and multifaceted nature of post-earthquake declines in QoL, reinforcing the necessity of integrated support strategies that simultaneously address both emotional and physical vulnerabilities.

The Wenchuan earthquake study, which dichotomized displaced survivors based on SF-36 population norms and examined associated factors using multivariable analysis, had a design highly comparable to ours. A holistic comparison of findings revealed that both studies observed a decline in all SF-36 domain scores relative to national standards, with similar risk groups identified. Among the shared independent variables, female gender and older age were associated with an increased risk of scoring below population norms, whereas higher education level and being single were protective factors. However, differences emerged in the domains influenced by these factors. Additionally, the Wenchuan study primarily focused on social support. In contrast, hospitalization and loss of relatives, which were assessed in our study, were not included in that analysis (7). Likewise, the Chi-Chi earthquake study used multiple regression analysis to examine factors associated with QoL measured by SF-36. Consistent with our findings, older age, female gender, and the presence of physical illness were identified as risk factors across various domains. However, in contrast to our study, being married was found to be a protective factor (10). Although differences may exist between populations facing similar circumstances, the general trends and risk factors display notable similarities, which may be influenced by cultural context and resilience factors.

Conducting follow-up studies in the evaluation of QoL, risk factors, and recovery models is also crucial, as it allows for the examination of the temporal changes in the effects of these factors and the identification of new determinants. Additionally, qualitative research approaches, such as in-depth interviews and focus groups, can provide a deeper understanding of the experiences of affected individuals. The combination of quantitative and qualitative methods will offer a more comprehensive perspective on post-earthquake quality of life (24).

Several potential limitations of our study should be considered. One of the limitations of our study is that, due to its cross-sectional design, the ability to assess causality is limited. Secondly, as the data were self-reported, potential biases such as recall bias and social desirability bias may have influenced the results. Additionally, as only one prefabricated housing area was selected for the study, the findings may not fully represent the broader population of earthquake survivors in the region, limiting their generalizability. Furthermore, while no formal sample size calculation was performed, the sample size of 334 participants represents approximately 82.9% of the estimated target population, which provides reasonable representation for the study. Lastly, our study did not account for certain potential confounders, such as pre-existing health conditions (e.g., chronic diseases) and psychological factors (e.g., post-traumatic stress disorder, depression, anxiety), which are known to influence QoL. Individuals with such conditions may have had lower baseline QoL, potentially confounding the observed associations. Despite these limitations, the study has several strengths. First, it is the only study to assess the QoL of earthquake survivors in Adıyaman following the February 2023 earthquakes. Second, the use of the SF-36, a well-established multidimensional assessment tool, enables a comprehensive evaluation of various aspects of QoL. Finally, conducting multivariable analyses while considering the deterioration in QoL relative to general population norms provides a valuable perspective on the determinants of post-earthquake QoL, contributing to a more nuanced understanding of the factors affecting survivors.

## 5 Conclusion

QoL among earthquake survivors affected by the February 2023 earthquakes in Türkiye and residing in prefabricated housing in Adıyaman has notably deteriorated compared to general population norms. This decline has been particularly severe in certain dimensions of QoL. Our findings indicate that the risk of having QoL scores below the population average, as assessed by the SF-36 tool, varies across different factors, with a greater number of affected domains observed particularly among women, those who lost a relative, and those with a history of hospitalization due to the earthquake. Targeted interventions within broader societal recovery efforts remain a critical necessity, with a particular emphasis on prioritizing high-risk groups to ensure more effective support strategies. Strengthening social support mechanisms may play a key role in mitigating the negative impact of post-disaster conditions on quality of life. Longitudinal assessments of QoL in the affected population will be essential to monitor recovery progress and guide future policy and intervention efforts. Moreover, there is a need for comprehensive public health interventions that extend beyond immediate emergency response to promote sustainable community resilience and recovery. The fundamental role of disaster preparedness plans, including health-focused strategies, should be recognized in mitigating long-term adverse effects.

## Data Availability

The raw data supporting the conclusions of this article will be made available by the authors, without undue reservation.
